# Spectral Response and Wavefront Control of a C-Shaped Fractal Cadmium Telluride/Silicon Carbide Metasurface in the THz Bandgap

**DOI:** 10.3390/ma15175944

**Published:** 2022-08-28

**Authors:** Ana Bărar, Octavian Dănilă

**Affiliations:** 1 Electronic Technology and Reliability Department, University Politehnica of Bucharest, 060042 Bucharest, Romania; 2 Physics Department, University Politehnica of Bucharest, 060042 Bucharest, Romania

**Keywords:** fractal metasurfaces, terahertz bandgap, frequency selective surfaces

## Abstract

We report theoretical investigations on the spectral behavior of two fractal metasurfaces, performed in the 3–6 THz frequency window (5–10 μm equivalent wavelength window), under illumination with both linear and circular polarization state fields. Both metasurfaces stem from the same tree-like structure, based on C-shaped elements, made of cadmium telluride (CdTe), and deposited on silicon carbide (SiC) substrates, the main difference between them being the level of structural complexity. The simulated spectral behavior of both structures indicates the tunability of the reflection spectrum by varying the complexity of the tree-like structure.

## 1. Introduction

Metasurfaces are artificial, 2D structures with unique electromagnetic properties, which are obtained by repeating unit elements, named meta-atoms. By modifying its geometry, dimensions or the composition of its meta-atoms, the response of a metastructure can be changed in a variety of ways. Compared to metamaterials, their 3D counterparts, metasurfaces, are more cost-effective to build. They have lower losses and they are less bulky, which allows them to be integrated in other devices. One example of such an integration is the replacement of bulky refractive lenses with metalenses [1,2]. The size of the meta-atoms—along with the element geometric sizes, relative positioning in the meta-atom and materials chosen for the individual elements—dictates the spectral window in which the metasurface is responsive in terms of reflection, transmission or absorption. As a rule of thumb, in order for a structure to respond to a wavelength λ, the elements of the structure must be of a size $a\in \left(\frac{\lambda }{10};\frac{\lambda }{5}\right)$ [3]. Due to progress in deposition and etching technologies [4,5,6,7], metasurfaces with sensitivities over a wide range of electromagnetic domains have been developed and investigated [8,9,10], from structures with unique properties in the visible domain, such as wavefront shaping [11,12,13], polarization control [14,15,16], diffraction [17], wavefront manipulation [18,19,20], harmonic generation [21,22,23] and optical cloaking [24,25,26,27], to metasurfaces designed for gigahertz (GHz) and terahertz (THz) applications, such as frequency selective surfaces [28,29,30] and re-writable memory [31,32,33,34]. The THz domain has been of particular interest, due to attractive features such as small attenuation, strong penetration and wide instantaneous bandwidth, or due to its inclusion of characteristic frequencies of a great number of chemical and biological molecules, which render it suitable for a wide range of applications in the fields of imaging [35,36], high-speed communication [37], time domain spectroscopy and chemical/biological sensing [38,39,40,41]. Examples of metasurfaces developed for THz applications include a structure based on square-shaped slits on a silicon substrate which was demonstrated to have extraordinary optical transmission resonance at 0.3 THZ, which rendered it particularly suitable for detection of trace amounts of antibiotics [42]; and a label-free sensor, based on a thin metallic mesh with resonant transmission in the THz domain, demonstrated for protein detection [43]. A more recent development in the field of metastructures was fractal metasurfaces [44,45,46]. Unlike regular metasurfaces, these structures have their meta-atoms arranged in fractal patterns, which allows spectral broadening of the structure’s response [47,48,49]. An implementation of this feature is demonstrated in [50], where a fractal plasmonic structure whose broadband spectral response could be tuned by controlling the degree of its fractal complexity. A polarization-insensitive and wide-angle fractal metasurface, achieving triple-band absorption, was also investigated. Another broadband absorber was demonstrated in [47], in the form of a plasmonic metamaterial with a dendritic fractal structure. A reflective, multifunctional and multiband fractal metasurface, based on metallic split-ring elements, was also theoretically and experimentally demonstrated [49].

In this paper, we theoretically investigate the electromagnetic responses of two sample fractal metasurfaces, stemming from the same cadmium-telluride (CdTe) tree-like structure, based on C-shaped elements made of deposited silicon carbide (SiC) substrates. The reasoning for choosing these materials is that on the one hand, CdTe is a semiconductor which has been used in the implementation of THz bandgap sources, and on the other hand, SiC is a low-cost, environmentally-friendly material with next-to-zero toxic byproducts resulting from the technical process. We performed simulations on several metasurfaces with fractal configurations, in which our goals were firstly to determine the suitability of SiC for the construction of metasurface architecture, and secondly, to observe the influence of multiple C-shape fractal design on the spectral response of the metasurface. The reason behind this study was mainly to identify possible applications of the CdTe/SiC metasurface in the desired spectral window. The main difference between the two metasurfaces is in the degree of structural complexity. The spectral behavior of the two metastructures was simulated under electromagnetic radiation, in a frequency window of 3–6 THz, and with different linear and elliptical polarizations. The reflection and phase spectra of the two metasurfaces were compared to a standard C-shape reference structure, simulated under the same conditions.

## 2. Design Considerations and Simulation Conditions

**Figure 1:**
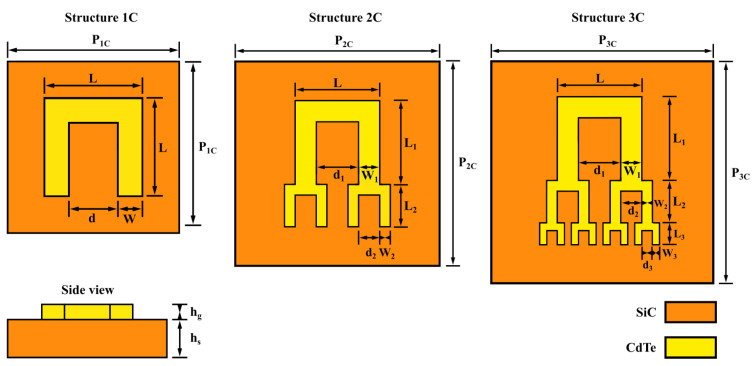
Layout of the fractal metasurface, together with the geometric parameters for the reference structure (1C), and the two C-shaped tree-like fractal structures (2C and 3C).

This paper proposes two structures based on the same C-shaped tree-like fractal geometry, made of cadmium telluride (CdTe), with different levels of complexity, each being built over a layer of silicon carbide (SiC). A reference structure, which consists of a single C-shaped element made of CdTe, over a layer of SiC, was also considered. Figure 1 illustrates the structures, with the notations of their respective dimensions, whose values are given in Table 1. The relative permittivity of CdTe was taken as $\epsilon_{r,CdTe}=10.39$, and the permittivity of SiC was taken as $\epsilon_{r,SiC}=10.8$. The reasoning for choosing this type of such a configuration is that the C-shape is the root geometry of split-ring resonators, which provide on-demand resonances depending on the size of the element. Additionally, the C-shape is a stackable geometry and is easy to include in a fractal architecture. In terms of electromagnetic material properties, we simplified the simulation considerably, without sacrificing accuracy, by considering all materials non-magnetic ($\mu_{r}=1$) and by neglecting the dispersion effects introduced by the imaginary parts of the relative permittivities. Additionally, in terms of frequency domain, we chose the terahertz bandgap as our desired spectral range based on the fact that the THz bandgap has been accessible only relatively-recently, and the exploration of different integrated-applications tailored to this spectral window is in its infant stages compared to the microwave and optical domains. Relevant applications that justify using these architectures in the THz bandgap include the development of integrated remote sensing systems for LIDAR-based industry and environmental applications [51,52], and THz-based molecular spectroscopy for cancer-related studies [53,54].

All three structures were simulated and studied using a commercial finite element time domain (FETD)-based simulation medium—COMSOL Multiphysics. The spectral behavior of the structures was simulated in a 5–10 μm (3–6 THz) window, with both linear and elliptical polarization, at various polarization angles. The FETD solution implements a standard Helmholtz equation on the resulting mesh, and provides the standard reflection and absorption coefficients at the output ports.

To have access at the polarization state of the input electric field amplitude, we used the Jones vector input of the form:

(1) $$\left(\begin{array}{c} E_{x}\\ E_{y}\\ E_{z} \end{array}\right)=\left(\begin{array}{c} \cos\left(\alpha \right)\\ \sin\left(\alpha \right)\cdot \exp\left(j\beta \right)\\ 0 \end{array}\right)$$

where α is the linear polarization angle, and β is the elliptical polarization angle. With the purpose of simulating the propagation, a rectangular box filled with air was assumed, with a propagation length L=15μm. The structure was placed at a coordinate y=5.5μm, with its director parallel to the long axis of the box. In order to ensure the periodicity of the medium, a perfectly matched layer (PML) was added at the top of the air box, at a coordinate $y_{PML}=11\;\mu$m. The input and output ports were added below the PML, at a coordinate $y_{input}=11\;\mu$m, and below the air box, at a coordinate $y_{output}=0\;\mu$m. In terms of boundary conditions, the walls of the air box were set to Floquet periodicity. The FETD solver was set to compute the frequency behavior of the structure, in the frequency interval 3–6 THz $\left(5–10\;\mu m\right)$, in the stationary regime, with a linearly polarized input electric field E, at different values of the linear polarization angle α. A similar computation was executed, with an elliptically polarized input electric field E, for different values of the elliptical polarization angle β.

## 3. Results and Discussions

**Figure 2:**
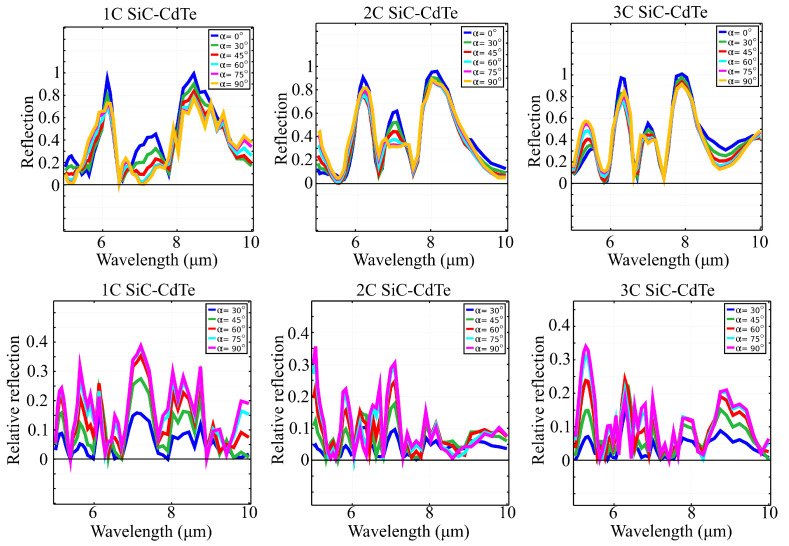
Absolute reflection (top row) and relative reflection (bottom row) spectra of structures 1C, 2C and 3C, for different linear polarization states of the input electric field. As the fractal layers are introduced, the smoothness of the response is increased. Additionally, for specific frequencies, the relative reflection coefficient is zero for all polarization states, which leads to zero-dichroism behavior as a function of polarization.

The obtained results are in agreement with previous investigations: the inclusion of fractal C-shape patterns in the architecture effectively broadened and smoothed out the spectral response of the window for all input polarization states. A possible explanation for this broadening is that the inclusion of multiple C-shaped elements of certain sizes excites surface plasmons at different resonance frequencies. As a result, with the increase in the fractal level, new resonance peaks are excited in the desired window. Separately, the symmetry of the fractal C-shapes excites dipolar and multipolar oscillations of the surface plasmon, which is known to offer a smoother envelope of the resulting response. In the case of CdTe/SiC fractal metasurfaces under study, we observed a strong response in reflection in terms of number of peaks and envelope smoothness. In the case of our configuration, the computed reflection spectra of all three structures, under a linearly polarized electric field, at different linear polarization angles α, are illustrated in Figure 2. The relative reflection was calculated as the absolute value of the difference between the reflection spectrum obtained at angle α and the reflection spectrum at reference angle $\alpha_{0}=0^{\circ }$. Table 2, Table 3 and Table 4 provide the main reflection peaks occurring in the reflection spectra of structures 1C, 2C and 3C, respectively. As the simulated structure becomes more complex, the number of reflection peaks occurring in the spectrum increases. At linear polarization angle $\alpha =0^{\circ }$, structure 1C yielded a total of four reflection peaks, at 5.2, 6.13, 7.42 and 8.45μm, of which two (5.2 and 7.42μm) disappeared gradually, as the value of angle α increased to $90^{\circ }$. Structure 2C yielded, at angle $\alpha =0^{\circ }$, a total of four reflection maxima (5μm, 6.2, 7.1, 8.17μm). One of these maxima (5μm) increased as the linear polarization angle α increased to $90^{\circ }$. Another one of these peaks (7.1μm) decreased as the value of angle α increased, but not to complete disappearance. At linear polarization angle $\alpha =0^{\circ }$, structure 3C has a total of five reflection peaks, at 5.57, 6.28, 7, 7.9 and 9.8μm; and two of these peaks (5.57μm and 9.8μm) shifted in wavelength and increased in amplitude as the polarization angle α increased to $90^{\circ }$. Another effect of structure complexity is the shift in wavelength of the reflection peaks, from one structure to another. This phenomenon is easily observed by following the shift in the three main reflection peaks occurring in the spectrum of structure 1C at 6.13, 7.42 and 8.45μm. These peaks also appear in the reflection spectrum of structure 2C, with similar or slightly higher amplitudes, but shifted in wavelength to 6.2, 7.1 and 8.17μm. The same three peaks also occurred in the reflection spectrum of structure 3C, at 6.28, 7 and 7.9μm. Additionally, in the case of structure 2C, a new reflection peak occurred at 5μm and was shifted to 5.57μm, in the spectrum of structure 3C. This shift in wavelength of the reflection peaks allowed the emergence of new peaks on the spectrum, as seen in the structures 2C (emergence of a peak at 5μm) and 3C (emergence of a peak at 9.8μm). An increase in structure complexity also led to a width narrowing of the reflection peaks. This effect is particularly evident, for example, in the case of the first significant reflection peak appearing the spectrum of structure 1C, at 6.13μm, where in has a full width half maximum (FWHM) of 0.75 at angle $\alpha =90^{\circ }$. For structure 2C, that same reflection peak, this time occurring in the spectrum at 6.2μm, has a FWHM of 0.58μm for angle $\alpha =90^{\circ }$. Lastly, for structure 3C, the reflection peak presents a FWHM of 0.51μm, at angle $\alpha =90^{\circ }$. The increased complexity of the structure also seems to reduce the amount by which the reflection peaks decrease in amplitude, as the linear polarization angle increases. As shown by the relative reflection calculated with respect to the values obtained for $\alpha =0^{\circ }$, the reflection peaks yielded by structure 1C decreased in amplitude with values as high as 0.4a.u.; structure 2C resulted in peaks that decreased with only 0.3a.u. The most significant decrease undergone by the reflection peaks of structure 3C was of 0.25a.u. Lastly, the increase in complexity of the structure led to a smoother reflection spectrum. Besides the main reflection peaks, the reflection spectrum of structure 1C also has several local peaks that increase in amplitude as the value of the linear polarization angle increases. These local variations in reflection tend to blur the main reflection peaks, this effect being most evident in the case of the peak encountered at 8.45μm. However, the reflection spectra of structures 2C and 3C have clearly defined peaks and minima of reflection.

**Figure 3:**
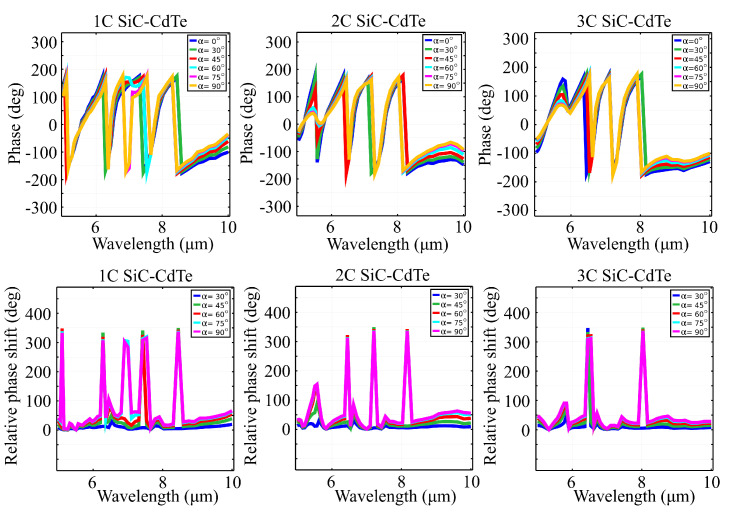
Absolute phase (top row) and relative phase shift (bottom row) spectra of the reflected wave after interaction with structures 1C, 2C and 3C at different input linear polarization states described by angle α. All configurations exhibit full 2π phase wavefront control and full phase-polarization dichroism for specific frequencies.

We also performed a study on the frequency responses of the metasurface for elliptic input polarization states. The study was justified due to the fact that the majority of sensors, especially those used in molecular spectroscopy, evaluate the changes in the elliptic input polarization after reflection. In the case of our metasurface, the computed phase spectra of all three structures, under a linearly polarized electric field, at different linear polarization angles α, are shown in Figure 3. Table 5, Table 6 and Table 7 indicate the main phase shifts that occur in these spectra. From the data, it can be seen that our metasurface exhibits full 2π phase coverage along the spectral window, which leads to the conclusion that full wavefront control can be achieved without prior phase preparation of the input field or by inducing any geometric phase by convenient global rotation of the C-shaped elements. The relative phase shift was calculated as the absolute value of the difference between the phase spectrum obtained at linear polarization angle α and the phase spectrum obtained at reference linear polarization angle $\alpha_{0}=0^{\circ }$. As the complexity of the structure increases, the number of phase shifts occurring in the spectrum of the structure tends to decrease. In the case of structure 1C, at angle $\alpha =0^{\circ }$, the phase spectrum presents four shifts, at 5.05, 6.2, 7.31 and 8.45μm. A fifth phase shift occurs at 7μm, as the angle α increases to $90^{\circ }$. For structures 2C and 3C, the phase spectra for angle $\alpha =0^{\circ }$ also present four phase shifts; however, the ones occurring before 6μm decrease substantially as the value of angle α increases. A common aspect of all three structures is a linear increase in phase taking place between 8.6 and 10μm. Structure 2C has a phase peak at 9.7μm, which increases with the value of angle α, but in the case of structure 3C, that peak is shifted to 8.6μm, followed by another region of linear increase in phase. At certain frequencies, the relative phase exhibits significant 2π jumps, which makes our metasurface exhibit full-phase polarization-based phase-dichroism. This selection criterion allows our metasurface to be used for polarization-based phase filters and phase-polarization wavefront sensors.

**Figure 4:**
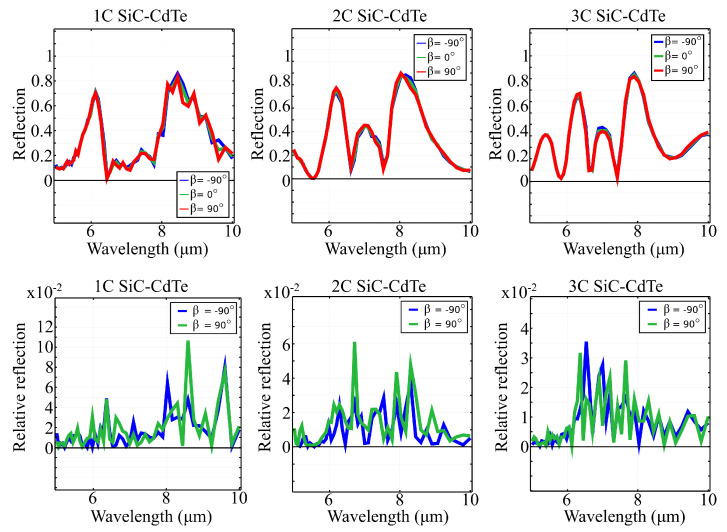
Absolute reflection (top line) and relative reflection (bottom line) spectra of structures 1C, 2C and 3C, under circularly-polarized electric field, at different values of the polarization angle β. The metasurface architecture is non-chiral, with relative reflection values below 10% across the spectral window.

The computed absolute and relative reflection spectra of all three structures, at different elliptical polarizations of the incident electric field, are shown in Figure 4. The relative reflection was calculated as the absolute value of the difference between the reflection spectrum obtained at polarization angle β and the reflection spectrum at reference polarization angle $\beta_{0}=0^{\circ }$. Based on the obtained data, it can be seen that our proposed metasurface architecture is relatively insensitive to the switch between circular and linear polarizations. The relative reflection of structure 1C is just above 0.1, at 8.6μm, and it decreases as the structure branches out. The maximum relative reflection of structure 2C is 0.6, at 6.7μm, and just above 0.34, at 6.6μm for structure 3C. Furthermore, the structures maintain their reflection peaks, regardless of the type of circular polarization. This insensitivity of the response as a function of the input polarization makes our metasurface ideal for reflection-based polarization controllers and reflection-based non-chiral wavefront controllers.

**Figure 5:**
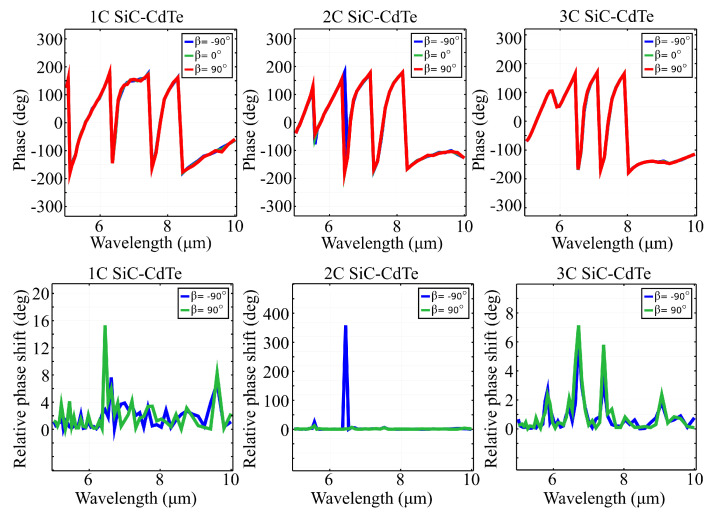
Absolute reflection (top row) and relative reflection (bottom row) spectra of structures 1C, 2C and 3C, under circularly-polarized electric field, at specific values of ellipticity angle $\beta =\pm 90^{\circ }$. The three architectures exhibit relatively low phase variations, with the exception of a small bandwidth where the 2C configuration exhibits a full 2π phase variation.

Just as in the case of linear polarization, our metasurface architecture exhibited a full 2π phase range across the spectral window of interest. Additionally, the phase variation is relatively low for circular polarization when referenced to the response obtained for an input linear polarization. The computed phase spectra of all three structures, under a circularly-polarized electric field, at different polarization angles β, are shown in Figure 5. The relative phase shift was calculated as the absolute value of the difference between the phase spectrum obtained at angle β and the phase spectrum obtained at reference angle $\beta_{0}=0^{\circ }$. In the case of structure 1C, changing the value of the elliptic polarization angle β induces very small modifications in the phase spectrum. The most notable occurred at 6.5 and 9.6μm. In the phase spectrum of structure 2C shows a significant phase shift transition in wavelength, from 6.5 to 6.6μm, indicated by a relative phase shift of $360^{\circ }$, for elliptic polarization angle $\beta =-90^{\circ }$. However, the phase spectrum of structure 3C is hardly changed by the variation of angle β, the most notable relative phase shifts being of merely $3^{\circ }$ at 6.9μm; $7^{\circ }$ at 7.4μm; $6^{\circ }$ at 7.7μm; and $3^{\circ }$ at 9μm, for both $\beta =-90^{\circ }$ and $\beta =90^{\circ }$. The results obtained further sustain that our metasurface is adequate for use as wavefront controllers and polarization controllers for input fields with linear and circular polarization states.

## 4. Conclusions

In this paper, we have presented simulations of the spectral properties of two CdTe/SiC fractal metasurfaces, under electromagnetic radiation in the terahertz domain, under illumination with a linear and circular polarization field in the terahertz gap. The metasurface architectures were obtained by repeating a CdTe C-shaped element over SiC substrate, in order to obtain tree-like structures with one and two layers of ramifications, respectively. Their spectral response may be tuned simply by increasing the number of ramifications in the structure, which allows obtaining a smoother spectral response in the desired frequency window, without fundamentally changing the geometry or the composition of the metasurface. Owing to the smoothness of the response offered by the fractal configuration, together with the insensitivity of the metasurface to circular polarization states, this cost-effective versatility allows our proposed metasurface architecture to be used in a wide range of terahertz devices, such as polarization-selective dichroic mirrors, polarization controllers, reflection-based terahertz sensors and wavefront controllers.

## Figures and Tables

**Table 1:** Geometric parameter values for the C-shapes in the configuration structures 1C, 2C and 3C.

Geometry	$P\;\left(\mu m\right)$	$h_{d}\;\left(\mu m\right)$	$L\;\left(\mu m\right)$	$W\;\left(\mu m\right)$	$d\;\left(\mu m\right)$	$h_{s}\;\left(\mu m\right)$
1C	25	0.5	15	2.5	10	0.5
2C	35	0.5	$L_{1}=15$ $L_{2}=7.5$	$W_{1}=2.5$ $W_{2}=1.25$	$d_{1}=10$ $d_{2}=5$	0.5
3C	45	0.5	$L_{1}=15$ $L_{2}=7.5$ $L_{3}=3.75$	$W_{1}=2.5$ $W_{2}=1.25$ $W_{3}=0.62$	$d_{1}=10$ $d_{2}=5$ $d_{3}=0.25$	0.5

**Table 2:** Peak values associated with the reflection spectrum of structure 1C, at linear polarization angles $\alpha =0^{\circ }$, $\alpha =30^{\circ }$, $\alpha =45^{\circ }$, $\alpha =60^{\circ }$, $\alpha =75^{\circ }$ and $\alpha =90^{\circ }$, where N/A stands for no reflection peak. These frequencies correspond to frequencies of minimal absorption.

Linear Polarization Angle α	Wavelength $\left(\mu m\right)$	Reflection Peak (a. u.)
$0^{\circ }$	6.13 7.42 8.45	0.95 0.45 0.99
$30^{\circ }$	6.13 7.42 8.45	0.94 0.45 0.99
$45^{\circ }$	6.13 7.42 8.45	0.71 0.23 0.83
$60^{\circ }$	6.13 7.42 8.45	0.69 0.17 0.79
$75^{\circ }$	6.13 7.42 8.45	0.64 N/A 0.61
$90^{\circ }$	6.13 7.42 8.45	0.75 N/A 0.59

**Table 3:** Peak values associated with the reflection spectrum of structure 2C, at linear polarization angles $\alpha =0^{\circ }$, $\alpha =30^{\circ }$, $\alpha =45^{\circ }$, $\alpha =60^{\circ }$, $\alpha =75^{\circ }$ and $\alpha =90^{\circ }$. These frequencies correspond to frequencies of minimal absorption.

Linear Polarization Angle α	Wavelength $\left(\mu m\right)$	Reflection Peak (a. u.)
$0^{\circ }$	5 6.2 7.1 8.17	0.13 0.9 0.62 0.96
$30^{\circ }$	5 6.2 7.1 8.03	0.17 0.8 0.52 0.9
$45^{\circ }$	5 6.2 7.1 8.03	0.24 0.75 0.44 0.88
$60^{\circ }$	5 6.2 7 8.03	0.32 0.75 0.38 0.87
$75^{\circ }$	5.05 6.28 7 8.03	0.41 0.78 0.33 0.88
$90^{\circ }$	5.05 6.28 7 8.03	0.44 0.81 0.31 0.89

**Table 4:** Peak values associated with the reflection spectrum of structure 3C, at linear polarization angles $\alpha =0^{\circ }$, $\alpha =30^{\circ }$, $\alpha =45^{\circ }$, $\alpha =60^{\circ }$, $\alpha =75^{\circ }$ and $\alpha =90^{\circ }$. These frequencies correspond to frequencies of minimal absorption.

Linear Polarization Angle α	Wavelength $\left(\mu m\right)$	Reflection Peak (a. u.)
$0^{\circ }$	5.57 6.28 7 7.9 9.8	0.34 0.97 0.55 1.04 0.44
$30^{\circ }$	5.45 6.28 7 7.9 9.8	0.36 0.82 0.49 0.97 0.42
$45^{\circ }$	5.39 6.28 7 7.9 10	0.41 0.75 0.45 0.94 0.42
$60^{\circ }$	5.39 6.36 6.9 7.9 10	0.49 0.75 0.44 0.91 0.45
$75^{\circ }$	5.33 6.36 6.9 7.9 10	0.55 0.81 0.43 0.91 0.47
$90^{\circ }$	5.33 6.36 0.8 7.9 10	0.57 0.85 0.4 0.91 0.49

**Table 5:** Phase transitions associated with the reflected wave across the phase spectrum in the case of structure 1C, at linear polarization states defined by $\alpha =0^{\circ }$, $\alpha =30^{\circ }$, $\alpha =45^{\circ }$, $\alpha =60^{\circ }$, $\alpha =75^{\circ }$ and $\alpha =90^{\circ }$.

Linear Polarization Angle α	Wavelength $\left(\mu m\right)$	Phase Transition
$0^{\circ }$	5.1 6.2 7.4 8.5	$200^{\circ }\;\mathrm{to}\;-200^{\circ }$ $200^{\circ }\;\mathrm{to}\;-180^{\circ }$ $180^{\circ }\;\mathrm{to}\;-180^{\circ }$ $180^{\circ }\;\mathrm{to}\;-180^{\circ }$
$30^{\circ }$	5.1 6.2 7.4 8.5	$200^{\circ }\;\mathrm{to}\;-200^{\circ }$ $180^{\circ }\;\mathrm{to}\;-180^{\circ }$ $175^{\circ }\;\mathrm{to}\;-180^{\circ }$ $180^{\circ }\;\mathrm{to}\;-180^{\circ }$
$45^{\circ }$	5.1 6.35 7.5 8.4	$200^{\circ }\;\mathrm{to}\;-200^{\circ }$ $200^{\circ }\;\mathrm{to}\;-150^{\circ }$ $180^{\circ }\;\mathrm{to}\;-180^{\circ }$ $175^{\circ }\;\mathrm{to}\;-180^{\circ }$
$60^{\circ }$	5.2 6.35 7 7.5 8.4	$200^{\circ }\;\mathrm{to}\;-200^{\circ }$ $180^{\circ }\;\mathrm{to}\;-160^{\circ }$ $200^{\circ }\;\mathrm{to}\;-200^{\circ }$ $200^{\circ }\;\mathrm{to}\;-110^{\circ }$ $180^{\circ }\;\mathrm{to}\;-180^{\circ }$
$75^{\circ }$	5.2 6.35 7 7.65 8.4	$200^{\circ }\;\mathrm{to}\;-200^{\circ }$ $180^{\circ }\;\mathrm{to}\;-160^{\circ }$ $190^{\circ }\;\mathrm{to}\;-190^{\circ }$ $200^{\circ }\;\mathrm{to}\;-110^{\circ }$ $180^{\circ }\;\mathrm{to}\;-175^{\circ }$
$90^{\circ }$	5.2 6.35 7 7.65 8.4	$200^{\circ }\;\mathrm{to}\;-200^{\circ }$ $180^{\circ }\;\mathrm{to}\;-160^{\circ }$ $200^{\circ }\;\mathrm{to}\;-200^{\circ }$ $200^{\circ }\;\mathrm{to}\;-110^{\circ }$ $190^{\circ }\;\mathrm{to}\;-190^{\circ }$

**Table 6:** Phase transitions associated with the reflected wave across the phase spectrum in the case of structure 2C, at linear polarization states defined by $\alpha =0^{\circ }$, $\alpha =30^{\circ }$, $\alpha =45^{\circ }$, $\alpha =60^{\circ }$, $\alpha =75^{\circ }$ and $\alpha =90^{\circ }$.

Linear Polarization Angle α	Wavelength $\left(\mu m\right)$	Phase Transition
$0^{\circ }$	5.5 6.4 7.2 8.2	$220^{\circ }\;\mathrm{to}\;-145^{\circ }$ $200^{\circ }\;\mathrm{to}\;-180^{\circ }$ $175^{\circ }\;\mathrm{to}\;-180^{\circ }$ $175^{\circ }\;\mathrm{to}\;-180^{\circ }$
$30^{\circ }$	5.5 6.4 7.2 8.2	$220^{\circ }\;\mathrm{to}\;-145^{\circ }$ $190^{\circ }\;\mathrm{to}\;-210^{\circ }$ $175^{\circ }\;\mathrm{to}\;-180^{\circ }$ $175^{\circ }\;\mathrm{to}\;-180^{\circ }$
$45^{\circ }$	5.5 6.4 7.2 8.2	$150^{\circ }\;\mathrm{to}\;-80^{\circ }$ $175^{\circ }\;\mathrm{to}\;-220^{\circ }$ $185^{\circ }\;\mathrm{to}\;-185^{\circ }$ $185^{\circ }\;\mathrm{to}\;-175^{\circ }$
$60^{\circ }$	5.5 6.4 7.2 8.2	$65^{\circ }\;\mathrm{to}\;-10^{\circ }$ $190^{\circ }\;\mathrm{to}\;-125^{\circ }$ $180^{\circ }\;\mathrm{to}\;-180^{\circ }$ $180^{\circ }\;\mathrm{to}\;-185^{\circ }$
$75^{\circ }$	5.5 6.4 7.3 8.15	$50^{\circ }\;to\;0^{\circ }$ $180^{\circ }\;\mathrm{to}\;-125^{\circ }$ $175^{\circ }\;\mathrm{to}\;-200^{\circ }$ $185^{\circ }\;\mathrm{to}\;-175^{\circ }$
$90^{\circ }$	5.5 6.5 7.3 8.3	nopeak $180^{\circ }\;\mathrm{to}\;-125^{\circ }$ $175^{\circ }\;\mathrm{to}\;-200^{\circ }$ $185^{\circ }\;\mathrm{to}\;-175^{\circ }$

**Table 7:** Phase transitions associated with the reflected wave across the spectrum in the case of structure 3C, at linear polarization states defined by $\alpha =0^{\circ }$, $\alpha =30^{\circ }$, $\alpha =45^{\circ }$, $\alpha =60^{\circ }$, $\alpha =75^{\circ }$ and $\alpha =90^{\circ }$.

Linear Polarization Angle α	Wavelength $\left(\mu m\right)$	Phase Transition
$0^{\circ }$	5.85 6.4 7.25 8.1	$160^{\circ }\;\mathrm{to}\;50^{\circ }$ $185^{\circ }\;\mathrm{to}\;-190^{\circ }$ $190^{\circ }\;\mathrm{to}\;-185^{\circ }$ $175^{\circ }\;\mathrm{to}\;-175^{\circ }$
$30^{\circ }$	5.85 6.5 7.25 8.1	$130^{\circ }\;\mathrm{to}\;50^{\circ }$ $190^{\circ }\;\mathrm{to}\;-190^{\circ }$ $190^{\circ }\;\mathrm{to}\;-190^{\circ }$ $180^{\circ }\;\mathrm{to}\;-175^{\circ }$
$45^{\circ }$	5.85 6.5 7.25 8	$110^{\circ }\;\mathrm{to}\;50^{\circ }$ $185^{\circ }\;\mathrm{to}\;-175^{\circ }$ $190^{\circ }\;\mathrm{to}\;-190^{\circ }$ $175^{\circ }\;\mathrm{to}\;-175^{\circ }$
$60^{\circ }$	5.85 6.6 7.25 8	$80^{\circ }\;\mathrm{to}\;40^{\circ }$ $200^{\circ }\;\mathrm{to}\;-110^{\circ }$ $175^{\circ }\;\mathrm{to}\;-190^{\circ }$ $175^{\circ }\;\mathrm{to}\;-175^{\circ }$
$75^{\circ }$	5.85 6.6 7.25 8	$75^{\circ }\;\mathrm{to}\;35^{\circ }$ $200^{\circ }\;\mathrm{to}\;-110^{\circ }$ $175^{\circ }\;\mathrm{to}\;-200^{\circ }$ $175^{\circ }\;\mathrm{to}\;-170^{\circ }$
$90^{\circ }$	5.85 6.6 7.25 8	$70^{\circ }\;\mathrm{to}\;35^{\circ }$ $200^{\circ }\;\mathrm{to}\;-110^{\circ }$ $175^{\circ }\;\mathrm{to}\;-200^{\circ }$ $175^{\circ }\;\mathrm{to}\;-170^{\circ }$
